# Brain imaging in children referred to pediatric neurology out-patients with headache

**DOI:** 10.1016/j.jped.2024.11.005

**Published:** 2024-12-07

**Authors:** Emine Ergül Sarı, Gonca Bektaş, Figen Palabıyik, Sadık Sami Hatipoğlu

**Affiliations:** a Bakırköy Dr. Sadi Konuk Training and Research Hospital, Department of Pediatrics, Istanbul, Turkey; b Bakırköy Dr. Sadi Konuk Training and Research Hospital, Department of Pediatric Neurology, Istanbul, Turkey; c Bakırköy Dr. Sadi Konuk Training and Research Hospital, Department of Pediatric Radiology, Istanbul, Turkey

**Keywords:** Brain imaging findings, Children, Headache

## Abstract

**Objective:**

Headaches are common in children and adolescents as well as in adults. Due to the fact that the primary medical concern for children presenting with headache complaints is the possibility of intracranial pathology, nowadays, imaging methods are frequently used in those patients.

**Methods:**

Retrospective data analysis was performed on the records of children who attended the Pediatric Neurology Outpatient Clinic between June 01, 2018, and December 01, 2018, complaining of headaches. Children who had a headache for longer than four weeks and had brain magnetic resonance imaging were included in the study. Brain MRI findings were classified as (1) headache-related and requiring definitive intervention, (2) possibly headache-related abnormalities, (3) headache-related abnormalities that did not require intervention, and (4) normal.

**Results:**

The 387 patients included in the study were between the ages of 2 and 17, with a median age of 10.5 years. Of the patients, 234 were female and 153 were male. The duration of the headache was a median of 12 months. According to brain MRI findings, 253 patients (65%) were in group 4, 79 patients (20%) were in group 2, 54 patients (14%) were in group 3, and 1 patient (0.3%) was in group 1.

**Conclusion:**

The probability of detecting significant abnormalities with brain MRI in children with chronic headaches with normal neurological examination is found to be low. Imaging methods should be kept in mind that they may be useful in diagnosis in selected cases.

## Introduction

Headache is quite common in children and adolescents as in adults. According to Abu-Arafeh's systematic review, the prevalence of headaches in children is 58.4%.^1^ Evaluation of systemic and neurological diseases in children presenting with headaches is important for correct diagnosis and treatment. Migraine is the most common cause of recurrent headaches in children and adolescents. Due to the fact that the primary medical concern for children presenting with headache complaints is the possibility of intracranial pathology,^2^ nowadays, imaging techniques are frequently applied to these patients. Non-invasive procedures like computed tomography and magnetic resonance imaging provide highly advanced anatomical and functional knowledge about cerebral structures. Particularly with MRI, hemodynamic alterations at the microvascular level can be seen. However, with the increase in MR imaging, in addition to the headache-related findings, coincidental findings unrelated to headaches are also detected.^3^

In the present study, the authors aimed to determine the diagnostic utility of brain imaging in children with chronic headaches and normal neurological examination.

## Materials and methods

A retrospective analysis of the records of children who attended the hospital's pediatric neurology outpatient clinic between June 1 and December 1 of 2018 complaining of headaches was performed as per the Hospital Clinical Research Ethics Committee's decision numbered 2019–06–07. All statistical analyses were performed using SPSS Statistics for Windows version 21.0 (IBM Corp., Armonk, NY, USA). Shapiro-Wilk test was used to check the normality of the data distribution. Continuous variables were expressed as mean, categorical variables were expressed as frequency and percentage. The Chi-square test (Fisher's Exact test) was used for comparisons between categorical variables. The Mann-Whitney U test was used to compare the parameters of the two groups. *p* < 0.05 was accepted as statistically significant.

Children who had a headache for more than four weeks and underwent brain magnetic resonance imaging were included in the study. Children with known neurological or psychiatric disorders and abnormal neurological examinations and patients who did not undergo MRI were excluded from the study. Demographic characteristics of the patients, duration of headache, and brain MRI findings evaluated by the Pediatric Radiology physician were recorded. MRI examination was performed using a 1.5 Tesla (Germany) MRI device. Brain MRI findings were classified as headache-related abnormalities requiring definitive intervention [1], possibly headache-related abnormalities [2], headache-related abnormalities that do not require intervention [3], and normal [4] (Table 1).

**Table 1:** Classification of neuroimaging findings.

Classification of brain imaging findings	Definition
Significant abnormalities	Associated with headache and requiring definitive intervention. Examples; acute cerebral infarction, acute cerebral edema, acute cerebral hemorrhage (subarachnoid, intra parenchymal or extra axial), neoplastic disease, hydrocephalus, and vascular abnormalities (for example, aneurysm or arteriovenous malformation)
Abnormalities possibly associated with headache	Probably headache related, may require definitive intervention. Examples; calvarium metastasis, acute or chronic sinusitis and abnormalities in the nasal cavity
Abnormalities unrelated to headache and not requiring intervention	Unrelated to headache or requiring no intervention. Examples; developmental venous anomaly, cerebral or cerebellar atrophy, subcortical infarction, old cortical infarction, and normal variants (e.g., cavum septum pellucidum, physiological calcifications)
Normal	

## Results

**Figure 1:**
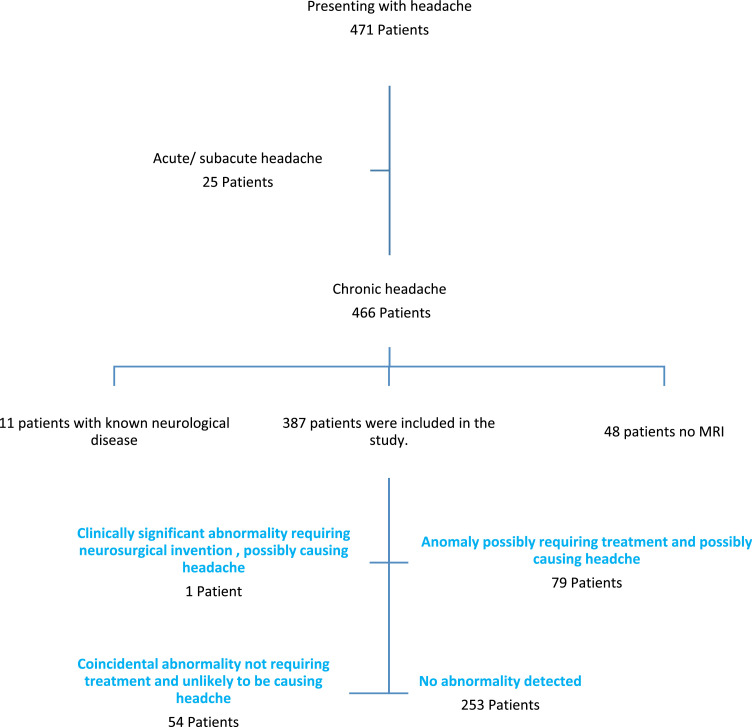
Distribution of patients according to brain imaging findings. (MRI, magnetic resonance imaging).

Data from 471 children presenting with headaches were analyzed. Eleven of the children with chronic headache and MRI were excluded due to their known neurological disorder. Twenty-five patients who had acute/subacute onset and 48 patients who did not have an MRI examination were excluded from the study. The ages of 387 patients included in the study were 2–17 years with a median age of 10.5 years (interquartile range [IQR] 9–14). Of the patients, 234 were female and 153 were male (females/males = 1.5). The duration of the headache was with a median of 12 months (interquartile range [IQR] 4–24). While 65% (*n* = 253) of the patients’ brain MRI findings were evaluated as normal, 14% (*n* = 54) had abnormalities related to headache and unrelated to headache and abnormalities not requiring intervention, 20% (*n* = 79) had definite intervention-related abnormalities that may be associated with headaches, and 0.3% (*n* = 1) had findings that required definite intervention and found to be related to headache (Figure 1).

**Figure 2:**
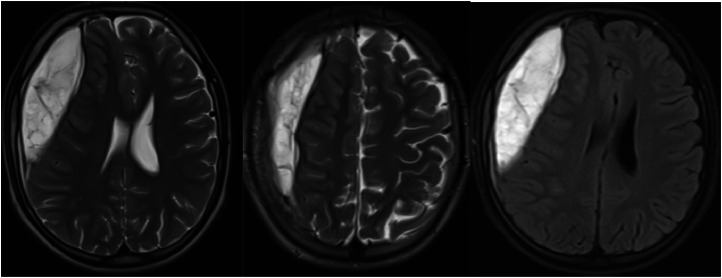
Brain imaging of the patient in the first group. Axial T2A/FLAIR sections show a subacute, chronic epidural, subdural hematoma in the right frontoparietal region, causing a shift in the midline.

A 15-year-old male patient who needed a decisive intervention for a headache-related symptom had a headache for two years as well as numbness in his left arm and tongue, which began with and continued after the headache and lasted for five to ten minutes. The neurosurgeon conducted a hematoma evacuation after a brain MRI revealed a subacute-chronic epidural-subdural hematoma in the right frontoparietal region that had shifted the midline (Figure 2).

Table 2 displays the MRI findings of patients who attended with headaches lasting for 4 weeks or longer

**Table 2:** MRI findings of patients presenting with headaches for 4 weeks or more.

MRI findings of patients presenting with headache for 4 weeks or more	
Clinically significant abnormality requiring neurosurgical invention, possibly causing headache	1
Anomaly possibly requiring treatment and possibly causing headache	**79**
Sinusitis Mastoiditis Frontal Osteomiyelitis Petrositis	73 4 1 1
Coincidental abnormality not requiring treatment and unlikely to be causing headache	**54**
Arachnoid cyst Adenoid vegetation Enlarged perivascular area Pineal cyst Neuroglial cyst Cavum septum pellicidum Partial empty sella < 5 mm hyperintense lesion on T2W/FLAIR sections Chiari type 1 malformation Choroidal fissure cyst Ventricular asymmetry Sinus retention cyst Polymicrogyria Thinning of the posterior corpus callosum Mega cisterna magna	15 11 4 3 3 3 2 2 2 2 2 2 1 1 1
No abnormality detected	**253**

## Discussion

Headache is the most common neurological symptom in childhood. Affecting more than 80% of children and adolescents, headache is a source of concern particularly for families and constitutes an important part of hospital admissions. Although headache is mostly caused by migraine and tension-type headaches, it may rarely be a sign of a life-threatening condition.^4^, ^5^, ^6^ For the diagnosis and evaluation of headache in this age group, detailed anamnesis, family observation and, in some cases, neuroimaging are needed.^7^, ^8^, ^9^ The American Academy of Neurology (AAN) and American College of Radiology (ACR) do not recommend neuroimaging for patients with primary headaches.^10^^,^^11^

Headache, which is seen equally in both sexes until adolescence, is more common in females starting from the adolescence period. Recurrent headaches were noted more frequently in females due to the commencement of menarche and pubertal development, according to a study by Gaßmann et al.^12^ Arruda et al. have found in their study that the complaints of headache in females were 2.5 times more common than in males.^13^ In the studied country, a study by Yılmaz et al. has demonstrated that 62% of the patients were female.^14^ In this study, the female gender was predominant, and the female/male ratio was found to be 1.5.

In hospital admissions, the majority of which are caused by migraine and tension-type headaches, if the neurological examination is normal, the neuroimaging efficiency is low; however, it is frequently performed due to the demands of the families and the risk of missing any underlying pathology. Although an application parameter for the imaging of children with headaches was published in 2002 by the Quality Standards Subcommittee of the American Academy of Neurology and the Practice Committee of the Child Neurology Society, it has been found that MR imaging was performed on 35–81% of cases in different studies.^15^, ^16^, ^17^ In a study of Yılmaz et al. conducted in 2014, cranial MR imaging was performed in 72.2% of the patients who attended the pediatric neurology outpatient clinic with the complaint of headache.^18^ In the present study, this rate was 89.8% and when the results were assessed, no pathology was detected in 65% of the patients. In the studies of Roy and Schwedt, it has been reported that 14% to 28% of pediatric patients with headaches who undergo neuroimaging have an abnormal finding.^15^^,^^17^ The fact that the study group consisted of patients who attended the pediatric neurology outpatient clinic may be the reason why this rate was found to be higher compared to other studies.

In 14% of the patients who had headache-related pathologies and did not require intervention, arachnoid cyst, adenoid vegetation, enlarged perivascular space, pineal cyst, neuroglial cyst, cavum septum pellucidum, partial empty sella, Chiari type 1 malformation, choroidal fissure cyst, ventricular asymmetry, sinus retention cyst, polymicrogyria, thinning of the posterior corpus callosum and mega cisterna magna were observed. In the study of Alaee et al., high-signal white matter lesions have been found most frequently in the MRI findings of children with migraine.^2^ In the study conducted by Lewis et al. with 302 patients who had headache complaints, 3.7% of patients with migraine and 16.6% of patients with chronic headache were found to have headache-related or unrelated (sinusitis, arachnoid cyst, Chiari I. malformation, vascular malformation, etc.) pathological findings; however, no surgical intervention was required in any patient.^19^ In the study of Dangouloff-Ros et al., most commonly cysts were found in MR imaging with a rate of 2.3%.^20^ In the MRI evaluation of patients diagnosed with idiopathic recurrent headache by Wöber, it was observed that most of the pathological findings detected in 17.7% of the patients were unrelated to headache.^21^ In this study, while cysts were discovered at a rate of 0.6% in the entire study group, 46% of the pathologies (*n* = 54) not associated with headaches that did not require intervention were observed.

In 79 patients included in the study, abnormalities, possibly related to headache, which may require definitive intervention, were detected, and most of them (92.4%) were sinusitis. In the study of Ceylan et al., patients with headache complaints have been evaluated with cranial computed tomography and the most common pathological finding has been found to be sinusitis with a rate of 17.4%.^3^ In the study of Alehan, sinusitis was the most common finding, and the diagnostic value of imaging methods was calculated as 14%.^16^ In the study of Bruton and Kan, sinusitis findings have been found in approximately 60% of the patients who attended with headaches.^22^^,^^23^ Although the most common finding of sinusitis was found in the studies of Dao & Qubty and Gürkaş et al., it has been emphasized that these findings caused anxiety in families.^4^^,^^8^

In the present study, only one patient (0.3%) had findings that required definite intervention related to headache. In that patient's brain MRI, the subacute-chronic, epidural-subdural hematoma was observed in the right frontoparietal region, which caused a shift in the midline. In the study in which Cain et al. evaluated the MRI of children and adolescents who attended the emergency room with headache, intracranial hemorrhage was found in two of 294 patients and an abscess in one.^24^ In Glatstein's study, one patient was diagnosed with multiple sclerosis (MS), while in Dangouloff-Ros's meta-analysis, 9 patients had asymptomatic tumors (4 low-grade glioma, 1 neuroepithelial dysembryoplastic tumor, 1 craniopharyngioma, 2 unspecified lesions, and 1 high-grade ependymoma).^7^^,^^20^ Some studies have listed red flags for neuroimaging in patients with headaches.^24^^,^^25^ In the study of Ahmed et al., significant brain abnormalities have been found in three of 386 MRI scans. Significant brain abnormalities were brain tumors, obstructive hydrocephalus, and tonsillar descent to C2.^25^ In Correnti et al.'s study, one patient who presented with a red flag had ischemia and one patient had astrocytoma.^26^

## Conclusion

Usually, anamnesis, a physical examination, and a neurological test are enough to determine the cause of childhood chronic headaches. Studies have shown that most of the findings in MRIs of patients presenting with headaches are benign. In accordance with the literature, the authors found that the probability of detecting significant abnormalities with brain MRI in children with chronic headaches and normal neurological examination was low (*p* > 0.05). Although imaging techniques shouldn't be asked as part of a normal examination for headache patients, it's important to keep in mind that they might be useful in selected cases to make a diagnosis.

## Informed consent

Informed consent was obtained from all individual participants included in the study.

## Ethics approval

A retrospective analysis of the records of children who attended the hospital's pediatric neurology outpatient clinic between June 1 and December 1 of 2018 complaining of headaches was performed as per the Hospital Clinical Research Ethics Committee's decision numbered 2019–06–07.

## Funding sources

The author(s) received no financial support for the research, authorship, and/or publication of this article.

## Author contributions

Conceptualization and study design and data analysis: Bektaş G, Data curation: Sarı EE, Palabıyık B, Writing and Language translate: Sarı E Supervision: Hatipoğlu SS.

## Conflicts of interest

The authors declare no conflicts of interest.
